# Editorial: Treatment and prognostic assessment of liver cirrhosis and its complications

**DOI:** 10.3389/fmed.2023.1359036

**Published:** 2024-01-10

**Authors:** Feifei Lu, Thierry Thévenot, Andrea Mancuso, Yiling Li, Lei Liu, Xingshun Qi

**Affiliations:** ^1^College of Medicine and Biological Information Engineering, Northeastern University, Shenyang, China; ^2^Liver Cirrhosis Study Group, Department of Gastroenterology, General Hospital of Northern Theater Command, Shenyang, China; ^3^Department of Hepatology, University Hospital of Besançon, Besançon, France; ^4^Dipartimento di Medicina Interna, ARNAS Ospedali Civico Di Cristina Benfratelli, Palermo, Italy; ^5^Department of Gastroenterology, The First Affiliated Hospital of China Medical University, Shenyang, China; ^6^State Key Laboratory of Cancer Biology and National Clinical Research Center for Digestive Diseases, Xijing Hospital of Digestive Diseases, Fourth Military Medical University, Xi'an, China

**Keywords:** liver cirrhosis, treatment, prognosis, pathogenesis, risk factor, complications

We are pleased to announce the success of the Research Topic entitled as “*Treatment and prognostic assessment of liver cirrhosis and its complications*” in the Frontiers, where a total of 10 high-quality papers have been published by 92 authors from Brazil, China, Germany, Italy, and Japan after internal editorial assessment, external peer review, and editorial decision processes. In this editorial, we summarize the contents of the 10 papers, which can be divided into two major sections, one about pathogenesis and risk factors, and another about prognostic assessment.

## Pathogenesis and risk factors of liver cirrhosis and its complications

### Liver cirrhosis

Zheng et al. from the Sun Yat-sen Memorial Hospital of Sun Yat-sen University, China, performed a meta-analysis to evaluate the association of the interferon-γ gene polymorphism rs2430561 and the tumor necrosis factor-α gene polymorphism rs361525 with the risk of liver cirrhosis. The currently available evidence demonstrated positive correlations of rs2430561 with alcoholic liver cirrhosis and hepatitis B virus related cirrhosis as well as that of rs361525 with hepatitis B virus related cirrhosis. This suggests the contribution of inflammation on cirrhosis. However, it should be acknowledged that the effect of the two gene polymorphisms on liver cirrhosis from other etiologies remains unclear.

### Osteoporosis in cirrhosis

Zhao et al. from the Shandong University of Traditional Chinese Medicine, China, reported the results of a bidirectional two-sample Mendelian randomization to clarify the causal association between primary biliary cirrhosis and osteoporosis by using the data from two public databases of genome-wide association studies, one about primary biliary cirrhosis, and another about osteoporosis. The investigators confirmed the causal effect of primary biliary cirrhosis on the risk of osteoporosis; by contrast and as expected, the presence of osteoporosis is not a causal factor for primary biliary cirrhosis. Generally speaking, this study should be the first to explore the causal relationship between them by using the Mendelian randomization method, and strengthens the necessity of routine screening for bone density in such patients.

### Hydrothorax in cirrhosis

Bai et al. from the Shandong University, China, conducted a retrospective study to evaluate the risk factors for hepatic hydrothorax in cirrhosis. It seemed that the severity of liver cirrhosis and portal hypertension, such as model for end-stage liver diseases, prothrombin activity, and portal vein diameter, as well as high-density lipoprotein cholesterol, would be closely associated with the development of this complication.

### Venous thrombosis in cirrhosis

da Cruz Renó et al. from the University of São Paulo, Brazil, evaluated the prevalence and risk factors of deep venous thrombosis and chronic venous insufficiency in outpatients with cirrhosis. Unfortunately, they did not find any variables which significantly correlated with deep venous thrombosis, but edema and orthostatism might be positively associated with the severity of chronic venous insufficiency. Zhang Y. et al. from the General Hospital of Northern Theater Command, China, attempted to analyze whether C-type lectin-like receptor 2 and galectin-1, two proteins involved in the activation and aggregation of platelets, were associated with the presence of portal vein thrombosis in hepatitis B virus related liver cirrhosis. Their association was significant, especially when portal vein thrombosis was more severe or extended to the superior mesenteric vein. Thus, *in vivo* studies should be warranted to validate their effects on the mechanism of portal vein thrombosis in cirrhosis.

## Prognostic assessment of liver cirrhosis and its complications

### General assessment

Gülcicegi et al. from the University of Cologne, Germany, reviews the recent advances regarding prognostic assessment of liver cirrhosis with an emphasis on current staging system for clinical course of liver cirrhosis, including compensated cirrhosis, stable and unstable decompensated cirrhosis, pre-acute-on-chronic liver failure, and acute-on-chronic liver failure grades 1–3. Besides, some novel scoring systems and biomarkers for liver cirrhosis have been reviewed as well as non-invasive approaches for assessment of portal hypertension.

### Decompensation in compensated cirrhosis

Saeki et al. performed a retrospective study to explore the prognostic impact of insulin-like growth factor, which can reflect liver function reserve, in patients with liver cirrhosis admitted to two medical centers in Japan. They found that a low insulin-like growth factor level should be an independent predictor of decompensation in compensated cirrhosis. Besides, a low insulin-like growth factor level was significantly associated with lower cumulative survival rates in compensated cirrhosis, but not decompensated cirrhosis.

### Hepatic encephalopathy in decompensated cirrhosis

Riggio et al. analyzed the impact of hepatic encephalopathy on the mortality and readmission of patients decompensated cirrhosis based on the data prospectively collected from 25 Italian referral centers. Multivariate competing risk analysis demonstrated that hepatic encephalopathy should be an independent predictor of death in cirrhotic patients. Meanwhile, patients with hepatic encephalopathy had a significantly higher probability of hospital readmission, and their readmission was primarily related to the recurrence of hepatic encephalopathy. These findings strongly indicated that patients with hepatic encephalopathy should be actively listed for liver transplantation. Similarly, in a retrospective single-center study, Zhang L. et al. from the Peking University People's Hospital, China, observed that hepatic encephalopathy recurrence was the most common cause for hospital readmission in patients with hepatic encephalopathy. Additionally, they identified neutrophil-to-lymphocyte ratio as a predictor of 30-, 90-, and 180-day hospital admission in such patients.

### Variceal rebleeding after in cirrhosis

Shi et al. selected patients with liver cirrhosis related to non-alcoholic steatohepatitis and hepatitis B virus infection who underwent hepatic venous pressure gradient measurement before and after transjugular intrahepatic portosystemic shunt at three medical centers in Chongqing, China. They identified a high hepatic venous pressure gradient of >17 mmHg after transjugular intrahepatic portosystemic shunt as an independent predictor for variceal rebleeding in non-alcoholic steatohepatitis related cirrhosis, but >20 mmHg in hepatitis B virus infection related cirrhosis.

Finally, we cordially thank all authors' contributions and editors' and peer-reviewers' efforts on the final publication of this Research Topic. Staff members from the Frontiers, especially Penerade Huang, should also be acknowledged because of their efficient and professional assistance on coordinating various issues involved in this Research Topic. We sincerely wish that the papers in the Research Topic can produce significant impact on clinical practice and academic research in future, and eagerly look forward to more high-quality submissions to the Research Topic Volume II in 2024.

## Author contributions

FL: Data curation, Formal analysis, Investigation, Validation, Writing—original draft, Writing—review & editing. TT: Formal analysis, Investigation, Validation, Writing—original draft, Writing—review & editing. AM: Formal analysis, Investigation, Validation, Writing—original draft, Writing—review & editing. YL: Data curation, Formal analysis, Validation, Writing—original draft, Writing—review & editing. LL: Data curation, Formal analysis, Validation, Writing—original draft, Writing—review & editing. XQ: Conceptualization, Investigation, Supervision, Validation, Writing—original draft, Writing—review & editing.

